# X-Linked Agammaglobulinemia Presenting as Neutropenia: Case Report and an Overview of Literature

**DOI:** 10.3389/fped.2021.633692

**Published:** 2021-06-28

**Authors:** Mosopefoluwa Lanlokun, Amanda Borden, Daime Nieves, Jolan E. Walter, Deborah Albright

**Affiliations:** ^1^Division of Allergy and Immunology, University of Pittsburgh Medical Center, Children's Hospital of Pittsburgh, Pittsburgh, PA, United States; ^2^Department of Medicine, Johns Hopkins All Children's Hospital, St. Petersburg, FL, United States; ^3^Division of Pediatric Allergy/Immunology, University of South Florida, St. Petersburg, FL, United States; ^4^Division of Pediatric Allergy and Immunology, Massachusetts General Hospital for Children, Boston, MA, United States

**Keywords:** neutropenia, immunodeficiency, immunoglobulin, case report, X-linked agamaglobulinemia

## Abstract

X-linked agammaglobulinemia (XLA) is an inherited immunodeficiency caused by mutations in the Bruton Tyrosine Kinase (*BTK*) gene. Marked neutropenia can be the initial abnormal laboratory finding in patients with XLA who are presenting with their first illness. The two cases presented herein support early consideration of evaluation for primary humoral immune deficiency in previously healthy male patients under the age of 12 months who present with neutropenia in the setting of infection shortly after passively acquired maternal antibody has sufficiently waned. Initial consideration of XLA (or other humoral immune deficiencies) in this particular population of young male neutropenic patients may afford the opportunity to avoid bone marrow biopsy in otherwise stable cases with similar presentations.

## Introduction

The differential diagnosis of neutropenia in the first year of life is broad and includes primary defects due to due to abnormal myeloid development, defective migration from the bone marrow, impaired neutrophil survival, and secondary (acquired) neutropenia often due to severe infections or other causes (1). The role of the neutrophil in preventing disease is highlighted by the fact that infants with severe congenital neutropenia often die before 1 year of age. In addition, in patients with HIV, neutropenia alone is known to increase risk of invasive bacterial infection and mortality (1). While the most common causes of neutropenia in the first year of life are acquired and can be due to viral infections, medications, or antibodies against neutrophils, neutropenia is also a significant finding in congenital disorders of bone marrow and primary immunodeficiencies (PIDs). There are examples of increased neutrophil death/consumption in several PIDs. In patients with adenosine deaminase deficiency, an inverse relationship has been identified between the accumulation of the toxic metabolites (adenosine and deoxyadenosine) and absolute neutrophil count, highlighting the role of neutrophils in disease progression. Additionally, immunodeficiencies that present later in life such as common variable immunodeficiency can also present with autoimmune cytopenias, including autoimmune neutropenia with circulating anti-granulocyte antibodies. X-linked agammaglobulinemia (XLA) and X-linked Hyper IgM syndrome are other primary humoral immunodeficiency disorders that may also present with neutropenia (1, 2). The estimated frequency of neutropenia in XLA, in particular, is ~22% (2–5). As a complete blood count is easily and often measured in children, it is not uncommon (11%) that the finding of neutropenia precedes the diagnosis of XLA (3). In this report, we aim to highlight the importance of consideration of additional immune evaluation in two patients with neutropenia who had delayed diagnoses of underlying XLA.

## Case 1

Our first case is an 8-month-old full term previously healthy male who was admitted to the pediatric intensive care unit with respiratory insufficiency and severe neutropenia, as a transfer from an outside hospital. The patient had initially presented to his pediatrician's office 3 days prior to admission with wheezing, trouble breathing, and fatigue. The pediatrician noted wheezing on exam and prescribed prednisolone and oral antibiotic (amoxicillin/clavulanic acid). His symptoms worsened, and he developed vomiting and diarrhea. Subsequent laboratory studies at an outside hospital revealed severe neutropenia with an absolute neutrophil count (ANC) of 0 cells/mm^3^ and marked lymphopenia with an absolute lymphocyte count (ALC) of 700 cells/mm^3^ (Total white blood cell count 1,500 cells/mm^3^; Table 1A). A chest X-ray showed no acute cardiopulmonary findings. Rapid testing for Epstein-Barr infection (monospot) and oropharyngeal streptococcal infection (rapid strep) were negative.

**Table 1:** Abnormal immunologic labs.

**(A) Complete blood count during current hospitalization**
**White blood cells 1,500 cells/mm^3^ (reference range 6–17.5)**
**Hemoglobin 11.3 g/dL (10.5–13.5)**
**Hematocrit 33 g/dL (33–39)**
**Platelets 354 cells/mm^3^ (156–369)**
**Absolute lymphocyte count 700 cells/m^3^ (4,000–13,500)**
**Absolute neutrophil count 0 cells/mm^3^ (1,000–8,500)**
**Quantitative immunoglobulins during current hospitalization**
**IgA <7 mg/dl (11–90)**
**IgG <33 mg/dl (217–904)**
**IgM <5 mg/dl (34–126)**
**Flow cytometry for lymphocyte characterization:**
**CD19 (B cells) 0 cells/μL (1,000–1,600)**
**CD3 (T cells) 389 cells/μL (2,400–3,300)**
**CD4 (T cells) 303 cells/μL (1,600–2,200)**
**CD8 (T cells) 58 cells/μL (820–1,600)**
**CD16/CD56 (NK cells) 92 cells/μL (270–1,100)**
**(B) Recurrent neutropenia**
**−3 weeks of age, during hospitalization for fever, rhinovirus infection ANC 900 cells/mm^3^ (reference range 1,000–8,500)**
**−8 months of age, during hospitalization for *H. influenzae* bacteremia ANC <200 cells/mm^3^**
**−8 months old, during hospitalization for fever, otitis media, pneumonia ANC 100 cells/mm^3^–Repeat ANC during hospitalization for otitis media, pneumonia: ANC 0 cells/mm^3^**
**Quantitative immunoglobulins during current hospitalization**
**IgA <6 mg/dl (reference range 11–90)**
**IgG <13mg/dl (217–904)**
**IgM 52 mg/dl (34–126)**

On transfer to our institution, a bone marrow biopsy was initially performed showing evidence of a hypocellular bone marrow with myeloid predominant hematopoiesis consistent with an immune mediated process without evidence of malignancy. Quantitative immunoglobulins resulted shortly thereafter and were undetectable (IgA: <7 mg/dl, IgG: <33 mg/dl, IgM: <5 mg/dl; Table 1A). Review of his newborn screen showed a normal number of T cell receptor excision circles. Further history obtained from the family suggested that the patient's maternal uncle had a history of recurrent infections and received “infusions” while the patient's parents and four siblings (male and female) were healthy. Additional immunologic testing (Table 1A) included flow cytometry which showed absent B cells and transiently decreased T cells and NK cells (CD19: 0 cells/μL; CD3: 389 cells/μL; CD4: 303 cells/μL; CD8: 58 cells/μL; CD16/CD56: 92 cells/μL) with normal fraction of naïve thymic emigrants (CD4+/CD45RA+/CD62L+ 80%). During this admission, blood culture grew *Pseudomonas aeruginosa*. The patient was treated with granulocyte colony stimulating factor (GCSF) for neutropenia along with Cefepime and Ciprofloxacin for Pseudomonas bacteremia. His absolute neutrophil (ANC) and lymphocyte (ALC) counts rebounded within 24 h of initiation of these therapies. Based on the family history and subsequent lab workup, XLA was suspected, and he was started on immunoglobulin replacement.

**Figure 1:**
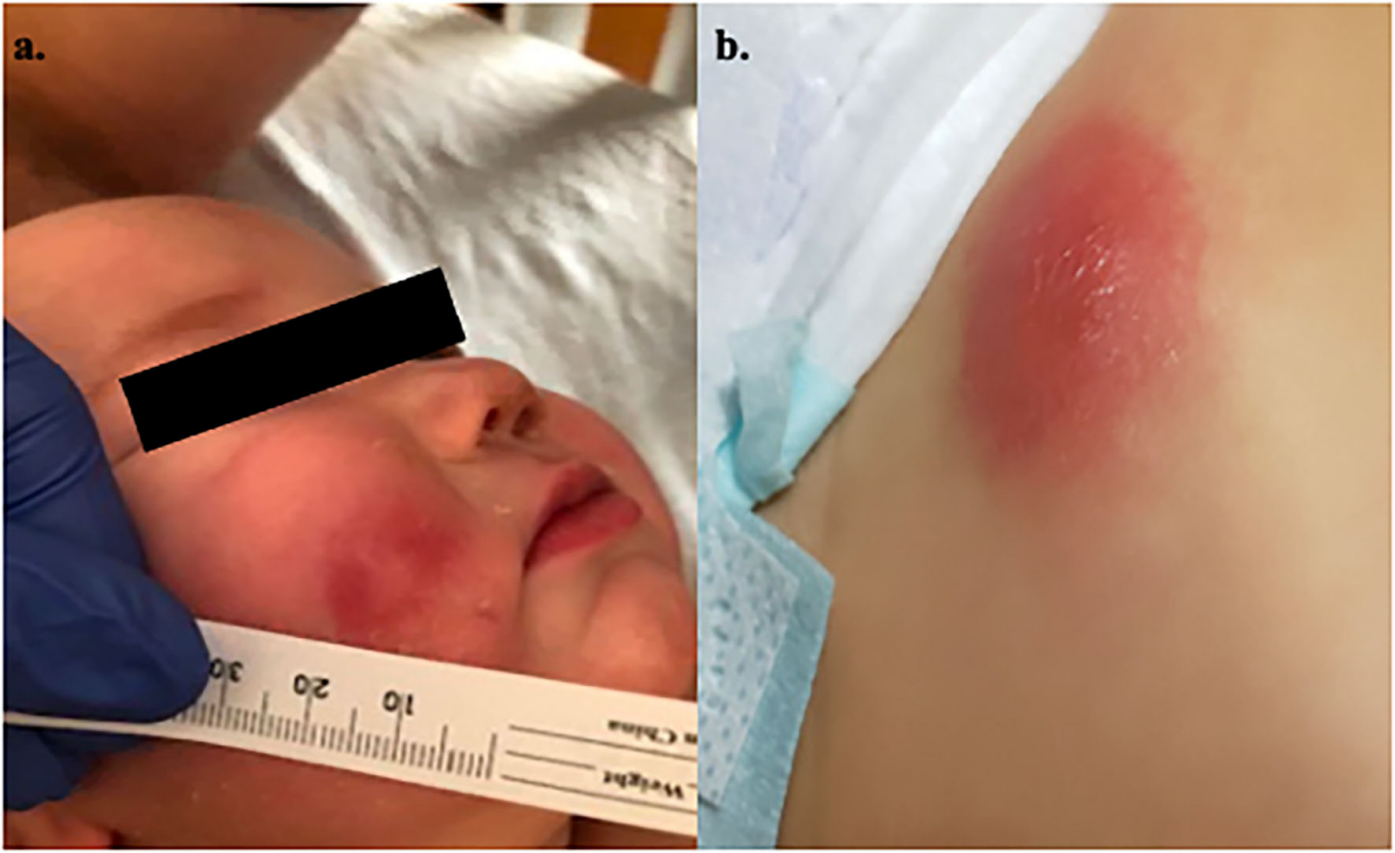
Patient 1's rash. **(a)** Rash on right cheek. **(b)** Rash on lower abdomen.

During this admission, he developed evolving skin lesions. While he presented with an erythematous, raised partially blanchable oval nodule (1.5 cm wide, 2 mm in depth) on his abdomen, he developed additional skin lesions after receiving filgrastim that were initially consistent with 3 mm vesicles on an erythematous base. A Varicella–Zoster virus DFA stain was negative and an initial skin biopsy of a lesion on the right leg was thought to be most consistent with an adverse drug reaction. He was discharged home on hospital day 9 with a 7-day course of ciprofloxacin for treatment of pseudomonas sepsis. Within 24 h of completion of this course he developed worsening skin lesions (on cheek and abdomen; Figure 1), low grade fever and acute redness and swelling of the right leg underneath his initial biopsy site. Magnetic resonance imaging of the leg showed evidence of pyomyositis and cellulitis in this area. A biopsy of another skin lesion on the left lower quadrant of the abdomen (Figure 1b) revealed deep dermal acute inflammatory exudate with necrosis, consistent with acute infectious abscess with focal vasculitis. The wound culture grew *P. aeruginosa* consistent with a diagnosis of ecthyma gangrenosum in the setting of Pseudomonas sepsis which was treated with an extended 4-month course of Ciprofloxacin.

Gene sequencing revealed a hemizygous pathogenic deletion of exon 19 (last exon) in the *BTK* gene, which has been previously reported in unrelated patients in the literature. The mutation results in the C′ terminal truncation of the BTK and confirms the diagnosis of XLA in our patient with neutropenia, agammaglobulinemia, and recurrent complicated pseudomonal infection.

## Case 2

Our second case is an 8-month-old male who initially presented with fever and ear discharge with subsequent evaluation revealing left upper lobe pneumonia and neutropenia with ANC 100 cells/mm^3^ (Table 1B). Further history revealed that he was born at term without complication but was hospitalized at 3 weeks of age for fever, and sepsis workup revealed rhinovirus and neutropenia (ANC 900 cells/mm^3^). Since that time, he had frequent episodes of otitis media and was off antibiotics for only 2 weeks between 4 and 6 months of age. He received his scheduled 2, 4-, and 6-month vaccines without complications and parents denied recurrent skin infections, eczema, failure to thrive, or chronic diarrhea. He has two older twin brothers who have had numerous ear infections requiring tympanostomy tubes and URIs but have never required hospitalization. Two weeks prior to his admission he was diagnosed with *Haemophilus influenzae* bacteremia with ANC < 200 cells/mm^3^ (Table 1B).

Total immunoglobulin levels were undetectable. The patient was referred to our Hematology service for evaluation of chronic neutropenia and underwent a bone marrow biopsy that was unrevealing. During his hospital stay, physical exam revealed a left ear canal obstructed with secretion but an otherwise healthy looking 8-month-old child with no vital sign abnormalities. His neutropenia was initially treated with GCSF. Repeat laboratory evaluation revealed: ANC 0 cells/mm^3^, IgG < 13 mg/dl, IgA < 6 mg/dl, IgM 52 mg/dl, absolute CD19 < 1 cells/μL, Table 1B), and vaccine titers showing no response to *Streptococcus pneumoniae* and *H. influenza* vaccinations. Therefore, the patient was started on immunoglobulin replacement therapy (IgRT) and neutropenia resolved.

In the absence of B cells, flow cytometry was obtained and revealed decreased but not absent intracellular expression of Btk protein in monocytes (MFI 3.55) relative to experimental control (MFI 7.3) which was suggestive of diagnosis of XLA in this male patient. Btk full gene sequencing was obtained revealing BTK, Intron 6, C.531-1G>A (splice acceptor), hemizygous—a variant that has been observed in individuals affected with XLA and resulted in low but not absent levels of the protein (6, 7). This confirmed the diagnosis of XLA in our patient with neutropenia, agammaglobulinemia, and recurrent infections.

## Discussion

In Case 1, the profound neutropenia was initially evaluated with bone marrow biopsy in investigation for an underlying hematologic pathology. It was not until after the bone marrow evaluation was completed that the key discovery of abnormal quantitative immunoglobulin levels was made prompting a further evaluation for defects in the humoral immune system. A confounding finding in this case was the marked concomitant reduction in CD4 T cells. While lymphopenia in patients with XLA is not unexpected due to the complete absence of B cells, diminished T cell counts are not commonly reported. In fact, T cell number and function are typically normal, as XLA is classically described as a disease that affects the humoral antibody compartment (8). The initial CD4 lymphopenia in this case in the setting of persistent respiratory symptoms and supplemental oxygen requirement led to short-lived concerns for a possible combined immunodeficiency with risk for *Pneumocystis jiroveci* pneumonia. Trimethoprim/sulfamethoxazole (TMP/SMX) was initiated for this reason, however, the subsequent normalization of the ALC and ANC along with elucidation of a vague family history of an immune disorder in the maternal uncle pointed to the likely diagnosis of XLA. TMP/SMX was eventually discontinued. This patient's skin rash was initially perplexing as it was originally attributed to an adverse drug reaction or possibly Sweet's syndrome given that most of his eruptions occurred after GCSF administration. The presence of an initial skin lesion prior to GCSF along with the acute worsening of the lesions within 24 h of completion of a 7-day course of ciprofloxacin led to a second lesional biopsy followed by culture which confirmed Pseudomonas as the etiology of the skin findings.

Case 2 also presented a clinical challenge as the recurrent ear infections were initially attributed by the primary care team to neutropenia. The absolute lymphocyte count in this case was normal for the patient which can sometimes be misleading in the consideration of B cell deficiencies such as XLA. Since B cell subsets only constitute 10–25% of the absolute lymphocyte count, the ALC can be within normal range even in the complete absence of the B cell compartment. Again, the key to the diagnosis in this case, as in Case 1, was the finding of abnormal immunoglobulin levels. While the definitive diagnosis of XLA in Case 2 was ultimately defined on genetic and molecular levels, earlier testing for quantitative immunoglobulin could have reduced the time between onset of infections, diagnosis of XLA and initiation of immunoglobulin replacement therapy.

Clinical history, absent to low serum immunoglobulins, and neutropenia should alert the astute practitioner to a potential diagnosis of XLA, especially in a young male patient. While Pseudomonas sepsis has been previously reported as an initial clinical manifestation of XLA, it has also been reported in disease states such as Wiskott–Aldrich syndrome, Chronic Granulomatous Disease, cyclic neutropenia, and other diseases involving neutrophil function and number (9, 10). In addition, it has been reported in previously healthy patients (9, 11). In case 1, the bone marrow biopsy was obtained prior to receipt of the results of the blood culture. In both cases, bone marrow biopsy for evaluation of neutropenia could have been avoided if investigation for XLA (with quantitative immunoglobulins and B cell enumeration) were initiated earlier. Absence of CD19+ B cells and/or decreased Btk protein expression by flow cytometry in monocytes are suspicious for XLA. Btk protein is normally present intracellularly both in B cells and monocytes, therefore the assay can be used even in full absence of B cells. Of note, 5% of *BTK* gene mutations permit Btk protein expression while abrogating function. To confirm the diagnosis, full gene sequencing can be completed. This also allows for comparison to population databases to determine whether specific mutations have been reported or previously found to be pathogenic.

## Management of XLA Patients With or Without Neutropenia

XLA patients are treated with IgRT and sometimes prophylactic antibiotics to prevent infections (7, 12). Immunoglobulin is administered intravenously (IVIG) or subcutaneously (SCIG) at varying intervals, usually every 2–4 weeks for IVIG and 1–14 days for SCIG. In addition, aggressive antibiotic treatment of acute infections is of utmost importance. The aim of treatment is to reduce infection, particularly invasive life-threatening infections, reduce risk of developing serious complications, including bronchiectasis, and maintain quality of life. If neutropenia is present, in selected cases granulocyte colony stimulation factor (GCSF) has been used. Interestingly, neutropenia in XLA is not commonly observed after initiation of consistent IgRT.

## Prognosis of XLA Patients With and without Neutropenia

Patients with XLA who succumb to invasive bacterial infection are thought to have such poor outcomes as a result of the combination of antibody deficiency and neutropenia. Kanegane et al. (2) suggest that immunoglobulin replacement therapy in patients with XLA may help to prevent subsequent episodes of neutropenia by reducing risk of infection. This association further underscores the importance of early diagnosis and initiation of therapy (2). The prognosis for patients with XLA has improved greatly as a result of earlier diagnosis, increases in antibiotic use and IgRT. If immunoglobulin replacement is started early and there is no persistent infection or malignancy, the prognosis can be good, and most patients can experience a near normal life.

The pathophysiology of neutropenia in patients with XLA is not yet well-understood. It has been postulated to be due to a variety of mechanisms including potential autoimmune mechanisms and/or lack of expression of *BTK* gene in myeloid cells. Another hypothesis is that the observed neutropenia may be due to a lack of Fc receptor activation of neutrophils and macrophages in the absence of IgG, further supporting why immunoglobulin replacement leads to an improvement in neutrophil counts (11). In addition, neutropenia has been observed in otherwise healthy patients with severe pseudomonal infections leading to the postulation that the neutropenia could be toxin mediated (13). In these cases, the neutropenia was transient and improved with appropriate antibiotic therapy. In both of our cases, before the definitive diagnosis of XLA, the observed neutropenia was initially treated with GCSF but ultimately resolved on IgRT.

Therefore, we conclude that recognition of neutropenia in male patients under 12 months of age in the setting of recurrent infections, sepsis, or soft tissue infection could be a sign of an associated humoral immunodeficiency. A low threshold for earlier evaluation with quantitative immunoglobulins may be of benefit. Furthermore, in our cases and as previously reported (2, 4, 11), appropriate antimicrobial therapy and/or IVIG replacement may, in many cases, lead to resolution of the neutropenia potentially obviating the need for further investigation with bone marrow biopsy and/or treatment with GCSF therapy.

## Data Availability Statement

The original contributions presented in the study are included in the article/supplementary material, further inquiries can be directed to the corresponding author/s.

## Ethics Statement

The studies involving human participants were reviewed and approved by University of South Florida (IRB protocol # Pro00025693 for JW). Written informed consent to participate in this study was provided by the participants' legal guardian/next of kin. Written informed consent was obtained from the minor(s)' legal guardian/next of kin for the publication of any potentially identifiable images or data included in this article.

## Author Contributions

ML and AB are co-first authors on this publication and wrote the manuscript and performed data interpretation. DN assisted with clinical data. JW and DA conceived the presented idea, reviewed and revised the clinical information presented, and supervised the findings of this work. All authors provided patient care, discussed the results, and agreed to the final manuscript.

## Conflict of Interest

JW has been consultant and speaker bureau member for Takeda, serves on advisory board for X4 Pharmaceuticals and CSL-Behring and he is the principal investigator on studies with Takeda, Momenta, Leadiant Biosciences, MustangBio, and CSL-Behring publication grants. The remaining authors declare that the research was conducted in the absence of any commercial or financial relationships that could be construed as a potential conflict of interest.
